# Design and Analysis of Cost-Efficient Sensor Deployment for Tracking Small UAS with Agent-Based Modeling

**DOI:** 10.3390/s16040575

**Published:** 2016-04-22

**Authors:** Sangmi Shin, Seongha Park, Yongho Kim, Eric T. Matson

**Affiliations:** M2M Laboratory, Computer and Information Technology, Purdue University, West Lafayette, IN 47907, USA; shin180@purdue.edu (S.S.); park708@purdue.edu (S.P.); kim1681@purdue.edu (Y.K.)

**Keywords:** sensor deployment, agent-based modeling, UAS tracking, distributed sensor network

## Abstract

Recently, commercial unmanned aerial systems (UAS) have gained popularity. However, these UAS are potential threats to people in terms of safety in public places, such as public parks or stadiums. To reduce such threats, we consider a design, modeling, and evaluation of a cost-efficient sensor system that detects and tracks small UAS. In this research, we focus on discovering the best sensor deployments by simulating different types and numbers of sensors in a designated area, which provide reasonable detection rates at low costs. Also, the system should cover the crowded areas more thoroughly than vacant areas to reduce direct threats to people underneath. This research study utilized the Agent-Based Modeling (ABM) technique to model a system consisting of independent and heterogeneous agents that interact with each other. Our previous work presented the ability to apply ABM to analyze the sensor configurations with two types of radars in terms of cost-efficiency. The results from the ABM simulation provide a list of candidate configurations and deployments that can be referred to for applications in the real world environment.

## 1. Introduction

### 1.1. Motivation and Goal

**Figure 1:**
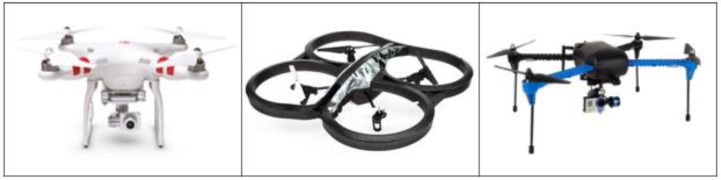
The three well-known commercial quadcopter unmanned aerial systems (UAS): DJI Phantom 3 [3] (Left); Parrot AR. Drone 2.0 [4] (Middle); 3DR IRIS+ [5] (Right).

While recent technologies of unmanned aerial systems (UAS) have benefitted many fields such as military, agriculture, and aerial photography, they are also associated with underlying threats, such as crash accidents by false-control or human error. Now with the release of small UAS for recreational and private purposes, UAS have become even more pervasive in the world. These commercial UAS, as shown in Figure 1, have gained popularity as they are relatively low-cost and easy to control with remote controllers for untrained users. However, they have brought new kinds of threats to security and safety of our society. For example, flying UAS near airports is strictly banned due to possible crashes with airplanes. Similarly, recklessly flying them in sports stadiums, schools, or near historic sites can injure people underneath or damage property. Also, privacy issues can occur when the camera equipped UAS record private or security areas. Moreover, there are concerns that small UAS could be involved in a terrorism. In January 2015, there was an incident in which a small commercial drone landed on the lawn of the White House, which posed a huge security issue [1,2]. Thus, there are high demands on developing a new technology that can detect and track small UAS to prevent such hazardous situations.

The goal of this research study is to design a distributed sensor network (DSN) that can detect small UAS in a public space. As an initial step, we aim to discover the cost efficient sensor configurations and deployments of the DSN in a designated area. Depending on the characteristics of the surveillance area and targets, sometimes deploying a couple of expensive long-range radars may be more efficient, while in some cases multiple cheap and short-range radars work better. In order to investigate such cases, we consider composing a sensor network with different types of sensors, and test which configuration works better. In terms of the coverage problem of DSNs, the ideal sensor deployment for detecting the unknown target in the surveillance area would be to cover the whole area under the detection range of the system. This is, however, often not feasible in a wide public place due to the high cost for the deployment of sensors. To deal with the ideal case considering the cost, we set constraints of the model, namely: (1) the amount of the budget cannot accommodate the full coverage of the surveillance area; and (2) higher-risk areas need to be more thoroughly monitored than other areas.

In our previous publication [6], we found a list of possible solutions with above constraints using Agent-Based Modeling (ABM). In order to extend the previous work, we conducted another ABM simulation with a different configuration (e.g., add another type of sensor, adjust the budget). In addition to this, verification of the ABM simulation was also performed.

### 1.2. Related Works

Wireless sensor networks (WSNs) or DSNs consist of a few, or even hundreds, of sensor nodes. Intelligent sensor deployment is important in DSNs as it can ensure the effective coverage area and decrease the need for excessive network communication for surveillance and tracking [7]. In particular, sensor deployment strategies for coverage problems [8,9] aim to obtain maximum coverage with the least number of sensors. Different strategies can be applied based on monitored areas, target characteristics, network deployment, and so on. For instance, there are grid based strategies [10], target coverage strategies [11], virtual force strategies for mobile sensor networks [12], and combined use of mathematical models for optimization under cost constraints [13]. Besides these methodologies, ABM has begun to be used in modeling distributed computer network systems as well, due to its growing complexity and the recent need for realistic modeling.

Agent-Based Modeling (ABM), or Agent-Based Modeling and Simulation (ABMS), is the modeling of a complex system composed of autonomous agents interacting with each other. Each agent in the system is defined with its static attributes and dynamic behavior rules. By integrating these encapsulated models to interact with each other through a simulation, the results show “patterns, structures, and behaviors that were not explicitly programmed into the models, but arise through the agent interactions” [14]. Thus, ABM has been applied to highly complex domains such as biology [15], social sciences [16], human systems [17], and geographical systems [18].

Because of its advantages, ABM has also been utilized to design and simulate WSN or DSN. For example, Niazi and Hussain [19,20] designed an ABMS framework called FABS (Formal Agent-Based Simulation) framework in order to design a realistic model and simulation of the WSN for monitoring a complex adaptive environment. The authors built a formal specification model to develop an agent-based model of the sensed environment, flocking of animals as an example, as well as the WSN with a random deployment of proximity sensors. Similarly, Hamzi and Koudil [21] proposed a platform for the design and simulation for WSN with an ABM approach by using abstract physical characteristics and dynamic behaviors of the network nodes. The platform is designed to support multiple target environments by enriching the translation functions for integrating the agent models and the target environment. Lin, Sedigh, and Miller [22] presented how to model an intelligent water distribution networks (WDNs) accurately with ABM. The authors used ABM to encapsulate diverse attributes of each independent semantic agent in the WDNs as well as capturing interactions among those heterogeneous agents in a distributed manner [22] Also, Batool *et al.* [23] presented guidelines for applying ABM to design and simulate different deployment types of WSN and advanced connectivity simulation for each type. The simulation results by their method showed the effectiveness of modeling complex WSN applications with an agent-based approach and also the expandability of adding mobility to sensor nodes (e.g., swarm robot sensors) compared to previous simulators. In addition, ABM can also be applied to an effective sensor power management strategy of WSN [24].

## 2. Design and Modeling

In order to use ABM in our problem domain, all the objects, namely sensors and UAS, should be appropriately modeled. The following subsections are the general settings of how we model such objects for the simulation.

### 2.1. Environment Design

#### 2.1.1. Surveillance Area

The place where people often choose to fly their UAS is a public and open space. A public park open to everyone has possibilities of UAS accidents which can cause damage to a person passing by. In this research study, we refer to Central Park, which is located in the downtown of New York City. Central Park is about 800 m wide and 4000 m long, so it is not easy to detect flying UAS in the public place by watching them using eyes.

#### 2.1.2. UAS

According to the classification guide of unmanned aerial vehicles (UAVs), which was published by the North Atlantic Treaty Organization (NATO) [25], UAVs can be classified into three categories: *Class 1, Class 2, and Class 3*. Among them, a configuration for the most common, recreational UAS which the public can easily buy, belongs to *Class 1,* Micro UAV. Recreational UAS are light-weight and small enough for people to carry one in their hand. UAS that are less than 2 kg and have normal operating altitude up to 200 feet above ground level (AGL) are classified as *Class 1* Micro UAVs.

In this research study, we assume that there is no limitation of space where an UAS user can control the UAS, so that an UAS can fly to any place in the designated area. The UAS is manually controlled in a random direction and speed, which are determined by an operator of the UAS. This means that the trajectory of the UAS is stochastic, unpredictable, and nonlinear. The details of modeling behavior rules for this nonlinear and unpredictable trajectory are described in Section 2.2.

#### 2.1.3. Sensors

We utilize three different types of radars in this simulation for a sensor system: (1) higher initial, operational, and maintenance cost with the widest detection range; (2) medium initial, operational, and maintenance cost with medium detection range; and (3) the lowest initial, operational, and maintenance cost with the shortest detection range. We randomly scatter these three different types of radars in the designated space, Central Park, to measure detection rate of the sensor configuration. We assume that all distributed radars can communicate to the base-station using their network communication ability to continuously track a target UAS. Also, the time loss of packet transmission is ignorable.

### 2.2. Agent-Based Modeling Approach

ABM makes it possible to model and simulate within a nonlinear, discontinuous, and stochastic system and environment [7]. The systems modeled in this research study are unpredictable and nonlinear flight patterns of UAS, and the heterogeneous types of sensors. Therefore, the ABM approach is applicable for these complex configurations to evaluate effectiveness of non-intuitive and unpredictable systems and environments. For instance, representing random flight patterns of recreational UAS with numerical models are limited because those patterns that come from the people who control the UAS cannot be numerically modeled. Moreover, the use of heterogeneous sensors increases the complexity of the system model. These difficulties make the DSN even harder to model and analyze with a general formulation. However, using ABM, which is a bottom-up design strategy that designs each entity and environment independently and enables us to observe the emerging results from their interactions, allows us to accommodate the aforementioned difficulties. The following subsections from Section 2.2.1 to Section 2.2.3 elaborate our methodology of the ABM approach based on the general settings in Section 2.1.

#### 2.2.1. Surveillance Area

The designated area is considered as a three dimensional grid, 800 m by 4000 m (3.2 km^2^), as the surface area is based on the size of Central Park, and the height limit of UAS flight is 61 m which equals the maximum operating altitude of *Class 1* Micro UAVs (200 feet) [25]. The minimum grid cell unit is 1 m by 1 m, and positions of radars and UAS are expressed in a Cartesian coordinate based on the unit grid cell. All UAS and radars operate within this designated area, and the surface of the area is assumed to be flat at this stage such that there is no obstacle restricting the line of sight of the sensors.

**Figure 2:**
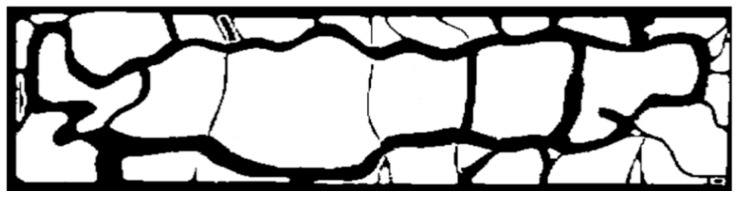
800 × 4000 pixel monochrome map of Central Park used as the target environment (**black**: roads/trails; **white**: lawn areas).

One of the purposes of this research study is to evaluate the effectiveness of sensor deployment within the designated area, as introduced in Section 1. To calculate the effectiveness of the sensor deployment, we assumed that the density of population in the park is higher on the roads or trails than the rest of the area, and thus the trails and roads need to be more significantly watched than the other areas. The map with the size of 800 × 4000 pixels, as shown in Figure 2, is image-processed into a monochrome map to distinguish the roads or trails from vacant lawn areas. We define *M* as a set of x, y coordination of pixels that are marked as roads or trails in the monochrome map of Figure 2. ($x_{min}$,$x_{max}$) and ($y_{min}$,$y_{max}$), in this case study, are (0, 800) and (0, 4000) respectively.

(1) $$M=\{\left(x,y\right)|x_{min}\leq x\leq x_{max},\;y_{min}\leq y\leq y_{max}$$

#### 2.2.2. UAS

The physical specification of *Class 1* Micro UAV was referenced for the simulation as shown in Table 1. The initial position of UAS in the simulation was randomly generated in each trial. Flight time for each UAS trajectory is 60 s with the sampling time of 0.5 s. When the UAS first takes off for 2 s, the x and y positions remain the same, and only the altitude changes. Flight patterns after the take-off are randomly generated, based on the behavior rule that the UAS randomly change their direction and speed within the limit that the dynamics of *Class 1* Micro UAV can handle at the current state of UAS (e.g., considering velocity, heading angle, altitude, *etc.*). Also, as discussed in Section 2.1.2, the probability that UAS appear in the surveillance area is the same for all locations, *i.e.*, the distribution of the occurrence is uniform.

#### 2.2.3. Sensors

Let *S* be a set of sensors (radars) used in each trial of the simulation, where i denotes the number of different types of sensors, j is the range of possible number of each sensor based on budget*,* budget is the total cost set for the simulation, and $cost_{i}$ is the cost of each radar’s type i.

(2) $$S=\{S_{1,1},\;S_{1,2},\;\cdots ,\;S_{i,j}\;|1\leq i\leq number\;of\;sensor\;types,\;1\leq j\leq \frac{budget}{cost_{i}}\}$$

Specifications of the three radar models used in the simulation are described in Table 2. As we discussed in Section 2.1.3, total cost includes initial, operational, and maintenance cost, which are proportional to the performance of the radar (e.g., detection range, resolution, *etc.*).

To set a total budget for this simulation, three cases are considered: (1) $48,000, the cost of deploying only radar 1 without any overlapping signals for 50% coverage of the surveillance area; (2) $53,750, the cost of deploying only radar 2 without any overlapping signals for 50% coverage of the surveillance area and (3) $23,700, the cost of deploying only radar 3 without any overlapping signals for 50% coverage of the surveillance area. Among these three costs, the smaller amount, $23,700, is used as a maximum budget, because this simulation aims to achieve the least expensive sensor deployment. The number of each type of radar is decided by the following equation, where *radar_1_*, *radar_2_*, and *radar_3_* represents the number of radar 1, radar 2, and radar 3 respectively:

(3) $$\begin{array}{c} 4,000\times radar_{1}+2,150\;\times radar_{2}+\;300\times radar_{3}\leq 23,700\\ \text{for}\left(0\leq \;radar_{1}\leq 5,\;0\leq \;radar_{2}\leq 11,\;0\leq \;radar_{3}\leq 79\right) \end{array}$$

The behavior rules of the radars can be formulized as follows. $d_{i,j}$ is the distance between the target (UAS) and the sensor $S_{i,j}$, where ($x_{u}$, $y_{u}$,$\;z_{u})$ and ($x_{S_{i,j}},\;y_{S_{i,j}}$,$\;z_{S_{i,j}})$ denotes the x, y, and z coordination of an UAS and the radar ($S_{i,j}$) each ($\text{in this study},\;z_{S_{i,j}}$ is always 0).

(4) $$d_{i,j}=\;\sqrt{{\left(x_{u}-\;x_{S_{i,j}}\right)}^{2}+{\left(y_{u}-\;y_{S_{i,j}}\right)}^{2}+{\left(z_{u}-\;z_{S_{i,j}}\right)}^{2}}$$

c(t)  defines the detection of the target, which means if one of the radars in the DSN senses the target within its detection range, it is determined as *detected* at time *t*. $r_{S_{i,j}}$ denotes the detection range of the sensor $S_{i,j}$_._ *t* is the duration of time and $t_{max}$ is the maximum value of it (here, $t_{max}$ = 60 s). The time *t* in this model is in discrete values with an interval of 0.5 (e.g., 0, 0.5, 1, ∙∙∙).

(5) $$c\left(t\right)=\{\begin{array}{l} d_{i,j}\leq r_{S_{i,j}},\;1\;\left(\mathrm{detected}\right)\\ d_{i,j}>r_{S_{i,j}},\;0\;\left(not\;detected\right) \end{array}\;|\;0\leq t\leq t_{max}\;\left(second\right)$$

### 2.3. Behavior Model Verification

**Figure 3:**
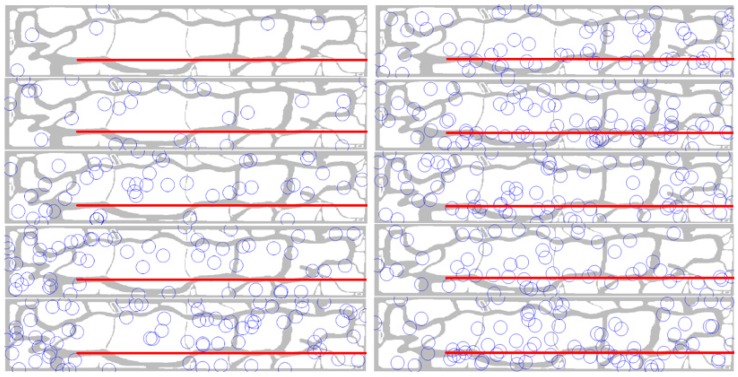
Behavior model verification: examples of results with low detection rates (**Left**) and high detection rates (**Right**) from the verification experiment (red line: UAS flight path, circles: positions of the radar).

Before implementing the simulation, the verification process was performed in order to inspect whether the behavior models described in Section 2.2 were implemented correctly, as designed. For the verification, minimized settings were used as follows to reduce the complexity: (1) UAS’s behavior rule: the target (UAS) flies straight from the coordination of (600, 800) to (600, 4000) with 10 m altitude (See the red lines on the bottom part of the maps in Figure 3); (2) Radars: only radar 3 was deployed and randomly changed its position 1000 times, as behavior rules are the same for all radars except for the detection range and cost. The number of radar 3 positions is decided by Equation (3). Other settings except for the above two were the same as in Section 2.2.

In each trail, the UAS detection rate was calculated by *an accumulated* *number of detection divided by the total number of samplings in a trial*. If the models in Section 2.2 were implemented correctly, it is expected that the best results (which have the highest detection rates) show a clear pattern of the radar positions that is mostly concentrated on the straight line of the UAS’s flight path (red line in Figure 3). On the other hand, the worst results (which have low detection rates) should be the opposite pattern of the radars that are scattered on the map, but do not overlap with the flight path.

As a result of this verification process (Figure 3), the radar positions with the highest detection rates show a clear pattern that the radars converge along with the flight path of the UAS. On the other hand, the list of worst results (detection rates less than 0.01%) shows the opposite patterns. Thus, the behavior models implemented in this research study can provide valid results in the further ABM simulations.

## 3. Implementation and Simulation

### 3.1. Implementation of the Models

**Figure 4:**
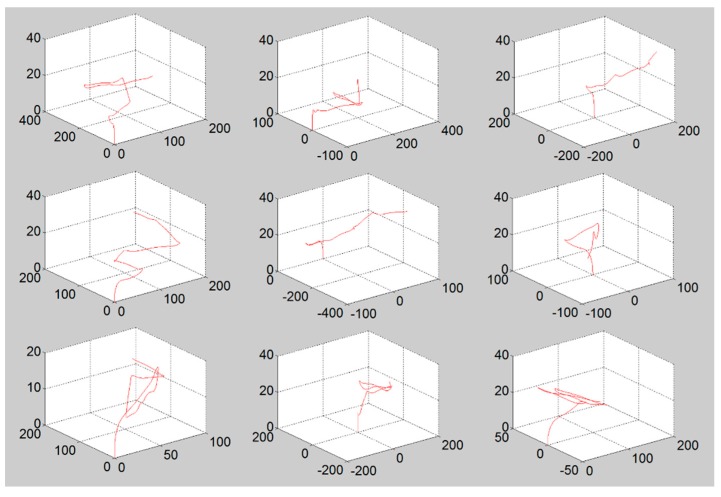
Examples of arbitrary flight patterns generated by Robotics Toolbox. (x, y, z in meters).

We implemented the aforementioned models into a simulation environment using MATLAB. In order to simulate a realistic UAS flight pattern on the basis of the model defined in Section 2.2.2, we utilized Robotics ToolBox [26]. The role of Robotics ToolBox in this simulation is to calculate UAS’s coordinates based on the UAS model shown in Table 1. This means that this toolbox gives the next coordinates of the UAS which are reachable at the next time frame without it falling down. Figure 4 shows several examples of the flight patterns among the 1000 samples that were used in the simulation. In the simulation, the data set of 1000 random patterns were used, which consists of x, y, and z coordinates at time t (every 0.5 s). The initial coordinates (x, y) of the UAS trial were uniformly selected within the range of surveillance surface in every trial while z coordinate always starts from zero, the ground.

### 3.2. Algorithm

Based on the budget, the simulation ran 1256 radar configurations and 300 random positions for each configuration. Each configuration was evaluated by 1000 different UAS flight patterns. The positions of radars as well as UAS were uniformly selected, as explained in Section 2. The detection was checked every 0.5 s as binary (as in Equation (5)) when at least one of the radars of the DSN detected a UAS. Also, because we put higher priority in the high risk area (roads/trails, which are the set as *M* in Equation (1)), there is a penalty of 0.5 when none of the radars of the system detected the UAS that is flying over *M* at time *t* (see *p(t**)*** in Equation (6)). The output of the algorithm is the *weighted detection rate* (Equation (7), where *k* is the number of UAS flights and $k_{max}$ is the maximum number of flights (here equal to 1000)), which deducted the penalty points at each critical failure of detection. This value was used to compare the performance of the deployment.

(6) $$p\left(t\right)=\{\begin{array}{c} \;c\left(t\right)=\;0\;and\;\left(x_{u},y_{u}\right)\in M,\;penalty\\ otherwise,\;0 \end{array}\;|\;0\leq t\leq t_{max},\;penalty>0$$

(7) $$\text{Weighted detection rate}=\frac{\sum_{k=1}^{k_{max}}\sum_{t=0}^{t_{max}}c\left(t\right)-p\left(t\right)}{t_{max}\cdot k\cdot \frac{1}{t_{interval}}}$$

## 4. Results and Discussion

The outcome of ABMS should be interpreted at the qualitative level based on the quantitative result of a simulation, because the output is varied depending on the degree of completeness in the input model from the real environment [17]. Thus, the results from this research study can be referred to in order to observe the general patterns, such as overall number of the radars and deployment outlines.

**Figure 5:**
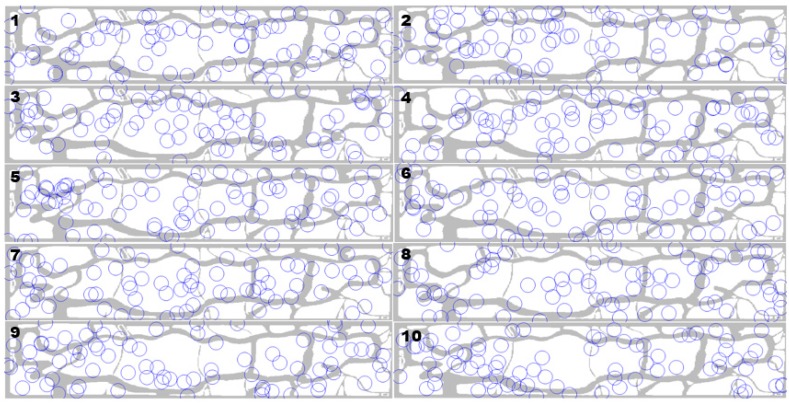
Sensor deployments of the best samples (ranking numbers on the top left). Circles represents each radar’s detection range.

Table 3 lists the top 10 results ordered by the weighted detection rate, among 50,240 trials. The deployment figures are displayed in Figure 5. Regarding the weighted detection rate in Table 3a, it is not an absolute value, but a relative value which is weighted by the penalty points mentioned in the previous section. This value represents how well the targets were detected by the DSN in the high risk area as well as in the other area.

In this case study, the result of the top one percent of samples (502 samples) ordered by the weighted detection rate indicates that, the performance of the system composed of only radar 3 with its nearly maximum numbers overwhelmed that of the system with other heterogeneous radar configurations. The configurations and deployment patterns in Table 3 and Figure 5 show higher values of detection rate in high risk areas (Table 3c) compared to the detection rate without penalties (Table 3b).

**Figure 6:**
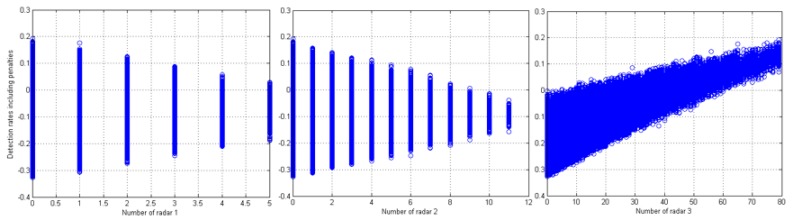
Detection rates including penalties (*y* axis) by number of each radar (*x* axis).

In terms of the number of radars in each type, Figure 6 shows that using a lower number of radar 1 and 2 resulted in higher detection rate, including penalties. In other words, the weighted detection rate increases as the number of radar 3 grows. For example, when using more than 60 radar 3s, the rates became all positive, meaning that use of a large number of radar 3 can guarantee the detection of UAS even in high risk areas based on the constraints that we applied. Negative values in Figure 6 are the results of getting more penalties than incidences of detection.

The results seem to be affected by the geographical characteristic of Central Park and the penalty points. For example, one of the characteristics of the area is the two thin and long trails that run along the long edges of the park. Thus, the patterns of each sensor position in Figure 5 are chained to each other following the trails. Also, as the width of the trails are thin enough to be covered by the smallest radar, which is radar type 3. The best results show that connecting a large number of radar 3 along the trail is more cost-efficient at detecting UAS that can harm people on the trails.

## 5. Conclusions

ABM is adopted in this research study to discover and analyze cost-efficient methods of sensor configurations and deployments for a small UAS tracking system in a public place. We conducted a case study on Central Park, New York, as a surveillance area. Also, to find cost-efficient methods, we set the constraints on the budget and gave penalties when failing to detect the target on the high-risk areas. The ABM approach of designing the simulation enabled us to model interactions between heterogeneous types of sensors and realistic flight patterns of commercial UAS. Verification tests for the behavior models were conducted to ensure the reliability of the modeling and simulation. The top results from the simulation show particular patterns of sensor configurations and deployments. These patterns from the simulation can be referred to for real world deployment possibilities.

This research study focused on the primary settings for the ABM simulation and thus the results may seem predictive at this stage. Further studies with more sophisticated settings from the real sensor models will be necessary to improve the reliability of the simulation. For instance, in this scope, we aggregated the numeric scales (e.g., operational cost and performance) to a cost of each sensor; however, using the ABM approach can still make the simulation extensible when the heterogeneous characteristics of each sensor are modeled separately for more realistic configurations, which are difficult to predict.

## Figures and Tables

**Table 1:** Attributes of UAS model based on *Class 1* Micro unmanned aerial vehicle (UAV) classification.

Specification	UAS Mode
Weight	4 kg
Maximum flight velocity	20.235 m/s

**Table 2:** Attributes of radar models.

Specification	Radar 1	Radar 2	Radar 3
Detection range (radius in m)	200	140	80
Total cost (U.S. dollars)	4,000	2,150	300

**Table 3:** Best 10 sensor configurations (ordered by (a) detection rate including penalties).

Ranking	No. of Radar 1	No. of Radar 2	No. of Radar 3	(a) Weighted Detection Rate	(b) Detection Rate Without Penalties	(c) Detection Rate in High Risk Area
1	0	0	79	0.1921	0.3783	0.4147
2	0	0	79	0.1889	0.3745	0.4070
3	0	0	75	0.1864	0.3639	0.4256
4	0	0	78	0.1818	0.3631	0.4330
5	0	0	79	0.1816	0.3637	0.4204
6	0	0	75	0.1809	0.3606	0.4219
7	0	0	76	0.1803	0.3705	0.4126
8	0	0	79	0.1788	0.3712	0.3795
9	0	0	79	0.1777	0.3656	0.4126
10	0	0	79	0.1776	0.3652	0.4210
